# Development and evaluation of a BCI-neurofeedback system with real-time EEG detection and electrical stimulation assistance during motor attempt for neurorehabilitation of children with cerebral palsy

**DOI:** 10.3389/fnhum.2024.1346050

**Published:** 2024-04-03

**Authors:** Ahad Behboodi, Julia Kline, Andrew Gravunder, Connor Phillips, Sheridan M. Parker, Diane L. Damiano

**Affiliations:** ^1^Department of Biomechanics, University of Nebraska Omaha, Omaha, NE, United States; ^2^Neurorehabilitation and Biomechanics Research Section, Rehabilitation Medicine Department, Clinical Center, National Institutes of Health, Bethesda, MD, United States

**Keywords:** associative learning, pediatric, NMES, transfer learning, motor training

## Abstract

In the realm of motor rehabilitation, Brain-Computer Interface Neurofeedback Training (BCI-NFT) emerges as a promising strategy. This aims to utilize an individual’s brain activity to stimulate or assist movement, thereby strengthening sensorimotor pathways and promoting motor recovery. Employing various methodologies, BCI-NFT has been shown to be effective for enhancing motor function primarily of the upper limb in stroke, with very few studies reported in cerebral palsy (CP). Our main objective was to develop an electroencephalography (EEG)-based BCI-NFT system, employing an associative learning paradigm, to improve selective control of ankle dorsiflexion in CP and potentially other neurological populations. First, in a cohort of eight healthy volunteers, we successfully implemented a BCI-NFT system based on detection of slow movement-related cortical potentials (MRCP) from EEG generated by attempted dorsiflexion to simultaneously activate Neuromuscular Electrical Stimulation which assisted movement and served to enhance sensory feedback to the sensorimotor cortex. Participants also viewed a computer display that provided real-time visual feedback of ankle range of motion with an individualized target region displayed to encourage maximal effort. After evaluating several potential strategies, we employed a Long short-term memory (LSTM) neural network, a deep learning algorithm, to detect the motor intent prior to movement onset. We then evaluated the system in a 10-session ankle dorsiflexion training protocol on a child with CP. By employing transfer learning across sessions, we could significantly reduce the number of calibration trials from 50 to 20 without compromising detection accuracy, which was 80.8% on average. The participant was able to complete the required calibration trials and the 100 training trials per session for all 10 sessions and post-training demonstrated increased ankle dorsiflexion velocity, walking speed and step length. Based on exceptional system performance, feasibility and preliminary effectiveness in a child with CP, we are now pursuing a clinical trial in a larger cohort of children with CP.

## 1 Introduction

Brain-Computer Interface (BCI) mediated neurofeedback training (BCI-NFT) involves using an individual’s brain signals during a motor task to trigger a robotic device or neuromuscular electrical stimulation (NMES), providing real-time sensory feedback and motor assistance. Thereby, BCI-NFT aims to fortify sensorimotor pathways, potentially fostering motor learning and neuroplasticity (Behboodi et al., 2022). BCI-NFT has been used effectively for motor rehabilitation in those with neurological disorders, especially individuals with stroke, as demonstrated in multiple systematic reviews (Monge-Pereira et al., 2017; Cervera et al., 2018; Bai et al., 2020; Kruse et al., 2020; Penev et al., 2023; Qu et al., 2024).

BCI-NFT systems typically utilize cortical activity associated with Motor Imagery (MI) or Motor Attempt (MA), as recorded by electroencephalography (EEG), to activate an external device. This device in turn induces stimulation or movement in the targeted body part, with or without additional visual feedback. MI involves mentally rehearsing movements without physical execution, and its neural substrates exhibit substantial overlap with the motor execution network (Bai et al., 2020). MI has been employed effectively as the method of choice in most BCI-NFT systems. In a randomized controlled trial (RCT), Ang et al. utilized an MI-based BCI with the MIT-Manus robot to train reaching tasks in 24 individuals post-stroke (Ang et al., 2010), while Frolov et al. employed a hand exoskeleton to train grasping in 74 individuals post-stroke (Frolov et al., 2017). Both studies revealed significant improvements in Upper Extremity (UE) Fugl Meyer Assessment (FMA) scores.

Despite disruptions in the sensorimotor loop in persons with neurological disorders, some motor planning or movement capabilities may be preserved. Thus, these individuals can employ MA, instead of MI, to activate an external device via BCI through attempted movements, even in cases of complete paralysis (Bai et al., 2020). In a controlled trial, Biasiucci et al. deployed an MA-based BCI-NFT to train wrist and finger extension of 27 participants post-stroke using NMES. They demonstrated significant improvements in UE-FMA scores, which notably lasted 9 months post-intervention (Biasiucci et al., 2018).

In MI-based rehabilitation, the central nervous system may need to actively suppress limb movement while the participant is visualizing the intended action, which requires learning and sustained concentration (Behboodi et al., 2022). Additionally, MI may be particularly challenging for young or cognitively challenged individuals (Bobrov et al., 2020). Even among healthy adults, mastering BCI systems through MI requires practice (Vidaurre and Blankertz, 2010; Zhang et al., 2020). Most participants have difficulty controlling the system initially and many, but not all, are able to gradually improve their BCI accuracy (Bai et al., 2020). Conversely, MA is considered more natural and verifiable. In neurofeedback training aimed at promoting or restoring motor functioning, choosing MA may improve outcomes by maximizing parallels between the brain-state used for control and the actual task, addressing concerns of task specificity that arise in MI paradigms, where maintaining consistent focus on the motor task is not assured (Behboodi et al., 2022). In a recent review, Bai et al. demonstrated that MI may be less effective for motor rehabilitation compared with MA (Bai et al., 2020).

Regardless of the brain state utilized (MI or MA), BCI-NFT systems typically employ classifiers to establish a mapping between brain activity and activation of external sensory stimuli, in real-time. Brain-state dependent simulation is another type of system for motor rehabilitation that uses data collected during a calibration phase to estimate the mean timing of the peak negative deflection prior to movement execution and employs that value to activate electrical stimulation or robotic assistance during motor training. While effective, these systems cannot be strictly considered BCI-NFT since the brain state, usually MA, is not detected in real-time, i.e., there is not an interface between the brain and the external device during motor training (Mrachacz-Kersting et al., 2019, 2021). In two RCTs utilizing brain-state dependent stimulation, including 22 (Mrachacz-Kersting et al., 2016) and 24 (Mrachacz-Kersting et al., 2019) participants with stroke, Mrachacz-Kersting et al. showed significant improvements in Lower Extremity (LE) motor function as measured by the LE-FMA, 6-meter Walk Test, and 10-meter Walk Test. The timing estimation was based on the peak negativity (PN), a prominent deflection of low-frequency movement-related cortical potentials (MRCP) (Mrachacz-Kersting et al., 2016) calculated during calibration, and was used to trigger the NMES system during training in a feedforward manner. PN is associated with motor intent and is usually detectable within a window of 500 ms prior to movement execution (Mrachacz-Kersting et al., 2021).

The selection of an external device, normally NMES or a robot, may also impact rehabilitation outcomes in BCI-NFT. Bai et al., in a subgroup meta-analysis, demonstrated the statistical superiority of BCI-driven NMES over BCI-driven robots for rehabilitation (Bai et al., 2020). Behboodi et al., however, did not observe a difference in effectiveness between the two devices in a MA-based BCI-NFT review in neurological populations (Behboodi et al., 2022). NMES is thought to be effective in fortifying the sensorimotor loop during BCI training through a more robust engagement of proprioceptive components, such as muscle spindles, in comparison to robotic devices; thereby increasing movement awareness during motor training and enhancing corticospinal excitability (Hu et al., 2015).

To induce motor learning, BCI-NFT studies deploy operant conditioning and associative learning paradigms. Operant conditioning is the most prevalent learning paradigm in BCI-NFT. It centers on inducing alterations in brain activity patterns, providing users with meaningful feedback to modulate their neural responses and maximize a reward (desired movement), thereby learning new motor skills. For instance, in the RCT by Ramos-Murguialdy et al. on 30 participants post-stroke, the experimental group received NMES if their Mu (8–13 Hz) band power was maintained below a predefined threshold (Ramos-Murguialday et al., 2019). In associative rehabilitative NFT paradigms, an endogenous task-specific brain state is temporally linked to sensory feedback, in accordance with the principle of Hebbian association which states that when two neurons repeatedly activate synchronously, they become more likely to fire together over time. The main advantages of this paradigm are its intuitive nature, which requires minimal or no training for learning how to control the system, and its potential to reduce the physical training dose required for meaningful motor improvements (Mrachacz-Kersting et al., 2021), as evidenced by the reported enhancements in lower limb functionality after just 20 min (30 pairings of brain-state and sensory stimuli) (Mrachacz-Kersting et al., 2016). To create the requisite association for neuroplasticity, it is imperative that the sensory feedback coincides with the peak cortical activation during MA. Positive correlations between induced plasticity and detection accuracy as demonstrated by Niazi et al. (2012), underscore the importance of classification algorithms that can detect the target EEG features quickly and accurately.

Training a classifier involves an extensive calibration period, and not all systems can achieve sufficient accuracy. The lower the accuracy, the more likely it is that subjects will not achieve the desired result consistently, or they will receive inappropriate feedback even when performing the task correctly (Irimia et al., 2018). BCI performance, as quantified by classification accuracy (number of correct classifications/total detection attempts), and the prevalence of false positives, significantly influences operant conditioning and associative learning. False negatives impede reward strategies crucial for patient motivation, while false positives can reinforce unintended actions, potentially leading to incorrect and mal-adaptive associations. The selection of source localization techniques, EEG features, EEG channels, and classification algorithms profoundly impacts detection accuracy. In operant conditioning, simple thresholding algorithms (Kim and Lee, 2016), similar to that used by Ramos-Murguialday et al. (2019), or machine learning classifiers like support vector machines (SVM) and linear discriminant analysis (LDA) are used to detect event-related desynchronization (ERD) in the EEG sensorimotor frequency band (~8–30 Hz), with accuracy ranging from 70 to 85%. Notably, a commercially available MI-based BCI-NFT system, RecoveriX, utilizes a Common Spatial Filter (CSP) and an LDA classifier achieving a mean accuracy of 87.4% across sessions in distinguishing left from right hand MI in stroke (Irimia et al., 2018).

Most BCI-NFT systems utilizing associative learning rely on Movement-related cortical potentials (MRCP) as their primary EEG feature. MRCP are low frequency EEG signals that demonstrate a negative shift that starts approximately 1.5–2 s before movement onset and thus enable one to detect motor intent before muscle activation. The peak in this negative deflection typically occurs within 500 msec of movement onset as described in a recent review (Shakeel et al., 2015). This makes detection of MRCP particularly applicable to BCI applications as well as rehabilitation ones because these facilitate a more timely (nearly real-time) response from a robotic, prosthetic or electrical stimulation device that will control or assist a movement. These have also been used often for neurofeedback applications because the detection of motor intent before an imagined or attempted movement can be used to assist the limb movement and provide enhanced sensory input to the motor cortex at the time that the brain is most active. The inherent assumption is that the closer this input occurs to the motor imagery or voluntary attempt, the stronger the facilitation of neuroplastic changes.

Depending on whether the motor task is cued as was done here (i.e., participants are warned that they will soon be given the cue to move, and when that is given, they should move as quickly as possible) or self-paced (participant is given an auditory or visual signal letting them know that they can perform the task whenever they want after an at least 2 s pause), the timing of the start of the negative deflection may vary given the two paradigms and this pre-movement negative signal may be referred to as Contingent Negative Variation, Bereitschafts or Readiness Potential or the Peak Negativity (Shakeel et al., 2015; Mrachacz-Kersting et al., 2019).

To address the temporal variability of MRCP both within and across subjects which limits its predictability, efforts have been made to detect MRCP features like PN in real-time. Niazi et al. introduced a supervised detection method utilizing a matched filter (Niazi et al., 2013); however, the time-variable and uncertain nature of the EEG signals reduces the effectiveness of matched filters (Ren et al., 2014). Thus, Niazi et al. then proposed an optimal spatial filter and achieved a detection accuracy of 82.5% in healthy individuals (Niazi et al., 2013). Xu et al. implemented a manifold learning algorithm followed by LDA, achieving over 80% detection accuracy (Ren et al., 2014). Bhagat et al. used SVM and demonstrated 79 ± 18% accuracy and a 23 ± 20% false positive rate (Bhagat et al., 2020). Recent endeavors have explored the use of Deep Learning models like Multilayer Perceptron Neural Networks (MLP-NN) (Behboodi et al., 2022) and Convolutional Neural Networks (CNN) (Lawhern et al., 2018) in detecting MRCP features in healthy individuals. These models typically employ a larger number of electrodes (21 and 64, respectively) and require minimal pre-processing and spatial filtering to identify EEG features.

The encouraging outcomes of BCI-NFT for restoring motor function in stroke provide the impetus for extending this paradigm to other neurological disorders like cerebral palsy (CP). CP is the most commonly diagnosed child-onset motor disability, characterized by compromised selective motor control. The efficacy of BCI-NFT in individuals with CP remains understudied with only a few studies utilizing various paradigms. For instance, Bobrov et al. employed a MI BCI-driven hand exoskeleton for feedback, conducting training sessions with 14 children diagnosed with CP (Bobrov et al., 2020). Remarkable enhancements in hand function, as measured by FMA, were induced after 7–10 weeks of training. This BCI system also demonstrated effectiveness in stroke (Kotov et al., 2014; Frolov et al., 2017). Kim et al. conducted an RCT involving 18 children with CP, revealing that MI BCI-driven NMES for hand extension training might evoke a more bilateral sensorimotor rhythm in frontopolar regions compared to NMES alone, after 30 half-hour training sessions (Kim and Lee, 2016). However, this study did not report specific motor outcomes. In another study (Olsen et al., 2021), participants with CP showed enhanced performance of the non-dominant hand on a serial reaction time task following classic neurofeedback training which involved attempts to modulate alpha power, facilitated with visual feedback. There is a specific need for the integration of MA and associative learning without the necessity of consistent imagination of specific movements or attempts to up- or down-regulate a motor-related EEG frequency band. MA significantly enhances the feasibility and accessibility of BCI-NFT for young children or individuals with cognitive impairments.

Our goal here was to develop and implement a real-time EEG BCI-NFT device that could be utilized in CP and other neurorehabilitation populations. Based on our scoping review of existing BCI-NFT methodologies (Behboodi et al., 2022), we focused on two major technological challenges when designing our system. First, we aimed to match or ideally improve detection accuracy beyond what was reported in the literature to reduce the amount of inappropriate feedback delivered during training. Second, we aimed to optimize the timing of EEG detection of motor intent in relation to the activation of sensory feedback delivered via NMES, so that the stimulation assisted the movement and was delivered when the sensorimotor cortex was the most active during the motor task. Here we describe these and other key components of our system, with a summary of the rationale behind the design decisions for each. Finally, to validate that the system performed as intended for a child with CP and to provide preliminary data on its potential effectiveness, we present data for our first clinical study participant. Although the system can be adapted for a range of motor tasks, ours was customized to train ankle dorsiflexion active range and joint angular velocity in those with CP who demonstrated reduced selective ankle control.

## 2 Methods

We developed a BCI-NFT system based on an associative learning paradigm which aims to establish a Hebbian association between task-specific neural activity, during attempts to dorsiflex the ankle, and sensory feedback induced via application of NMES.

### 2.1 Description of our BCI-NFT system

**Figure 1:**
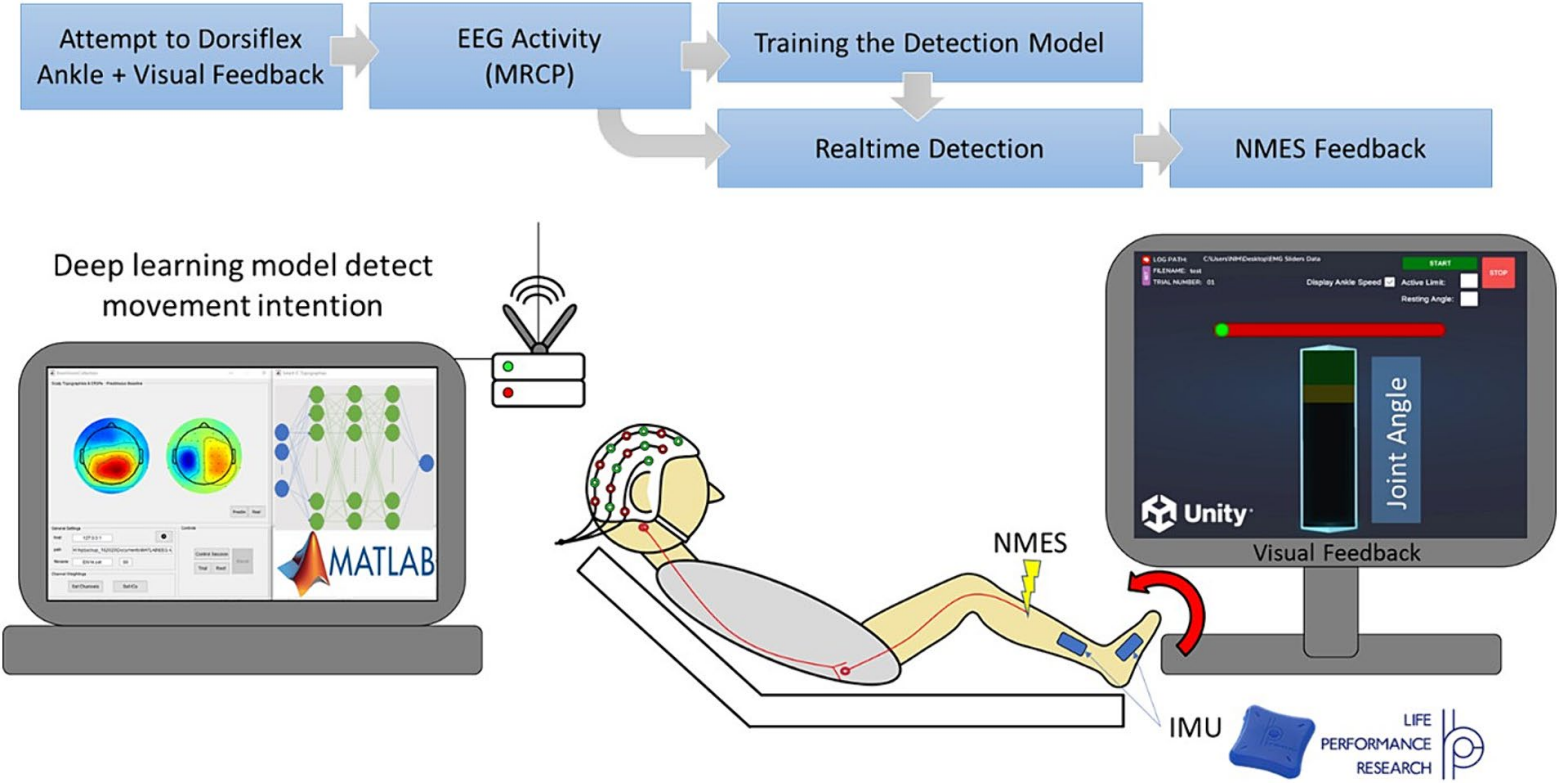
Diagram of our Brain-computer interface neurofeedback training (BCI-NFT) system which utilizes MRCP of the EEG signal to detect participant motor intent. The participant was tasked to dorsiflex the ankle at a specific time, cued by a graphic user interface (GUI), which also provided visual feedback of the ankle angle in real-time. EEG data were recorded using a 64-channel EEG system and streamed wirelessly to the BCI-NFT software using Stimulation Delivery. Upon detection of motor intent from EEG, the BCI-NFT system triggered a stimulator to apply assistive sensory feedback (NMES of the tibialis anterior muscle). To monitor ankle angle in real-time, two Inertial Measurement Units (IMUs) continuously streamed joint angle data to the visual feedback GUI in Unity.

Our BCI-NFT system utilized MRCP of the EEG signal (0.05–10 Hz) to detect the participant’s motor intent within a window of 500 ms prior to movement onset. The chosen frequency band was based on the MRCP range utilized by Mrachacz-Kersting et al. (2016) and Mrachacz-Kersting et al. (2019); however, other researchers may utilize an even more truncated frequency range for MRCP (Olsen et al., 2021). The participant was tasked to dorsiflex the ankle at a specific time, cued by a graphic user interface (GUI), which also provided visual feedback of the participant’s ankle angle in real-time. Once the BCI system detected the participant’s intention to dorsiflex, the NMES component was activated to stimulate the tibialis anterior (TA) muscle (Figure 1).

#### 2.1.1 Hardware

##### 2.1.1.1 Joint angle

To monitor ankle angle in real-time, we used two Inertial Measurement Units (IMUs), LPMS B2 series (Life performance Research, Tokyo, Japan). These IMUs facilitated the continuous streaming of joint angle data to the visual feedback GUI. The real-time calculation of joint angle was accomplished through a Unity interface developed by the IMU manufacturer.

##### 2.1.1.2 EEG acquisition

EEG data were recorded using a 64-channel active EEG system (Brain Products, Munich, Germany) at a sampling frequency of 500 Hz. EEG data were streamed wirelessly to the BCI-NFT software using the BrainAmp amplifier (Brain Products, Munich, Germany).

##### 2.1.1.3 Stimulation delivery

Upon detection of motor intent from EEG, the BCI-NFT system triggered a stimulator, via serial communication, to apply assistive sensory feedback (NMES to the tibialis anterior muscle of the more affected limb). The stimulator was a modified version of a Food & Drug Administration (FDA)-approved four-channel LG-8TM Elite Electrical Muscle Stimulator (LG Med Supply).

#### 2.1.2 Software

The software consisted of three primary components:

1. *Visual feedback GUI in Unity* (Unity Software Inc., San Francisco, CA): This component was responsible for providing task cues and real-time visual feedback of the participant’s ankle angle.
2. *Operating GUI in MATLAB* (MathWorks, Natick, MA): The MATLAB-based interface was used for configuring detection parameters and real-time execution of the BCI.
3. *LabStreamingLayer (LSL)*: LSL (Kothe et al., 2024) was utilized to synchronize data streams, including joint angle data, triggers from the visual feedback GUI, and EEG signals.

**Figure 2:**
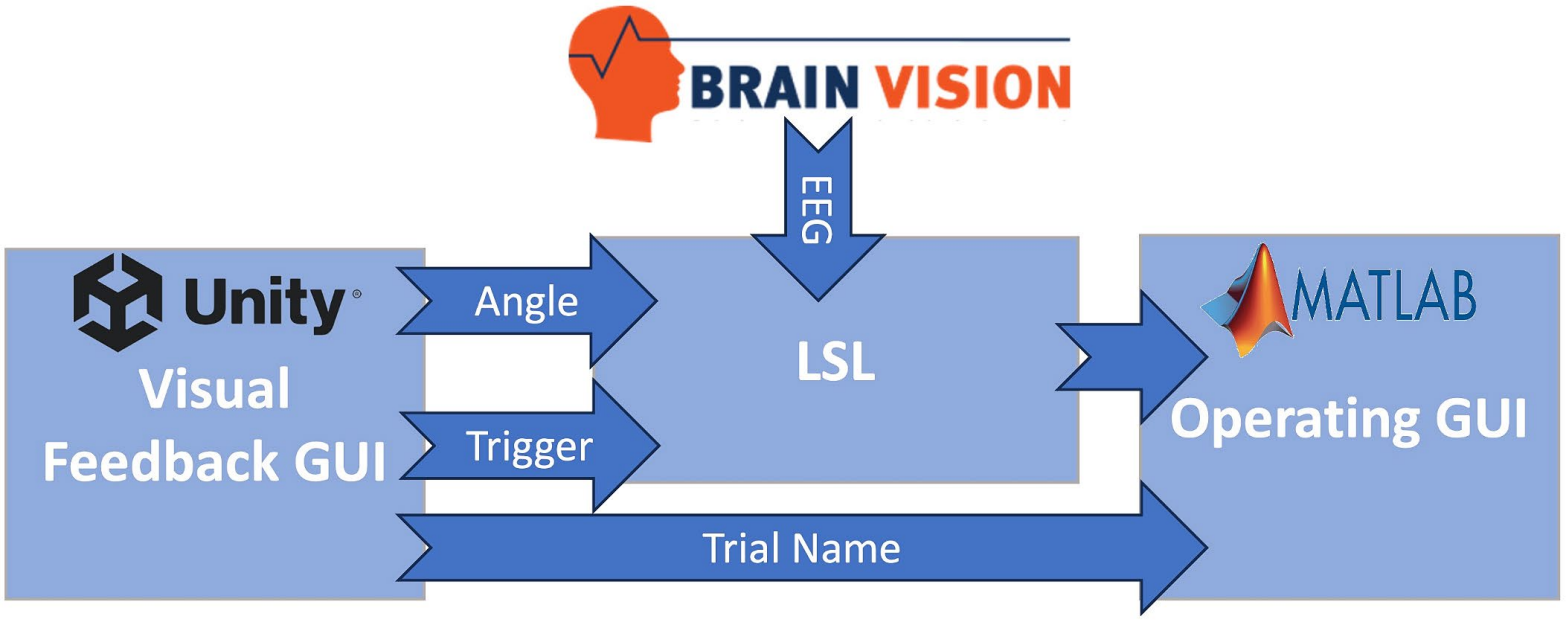
Information flow of the BC-NFT software. The visual feedback GUI transmitted trigger values associated with various task events (provided as visual cues to the participant) and ankle angle data to LSL. EEG was directly streamed into the Lab Streaming Layer (LSL) in real-time. LSL played a crucial role in synchronization and timestamping all data streams, i.e., EEG, ankle angle, and triggers. Subsequently, it transmitted the EEG and trigger streams to the BCI system in MATLAB.

Figure 2 is a visual representation of the data flow within the software. The visual feedback GUI transmitted trigger values associated with various task events (provided as visual cues to the participant) and ankle angle data to LSL. EEG was directly streamed into LSL in real-time using an LSL plugin provided by Brain Products (Munich, Germany), the EEG system manufacturer. LSL played a crucial role in synchronization and timestamping all data streams, i.e., EEG, ankle angle, and triggers. Subsequently, it transmitted the EEG and trigger streams to the BCI system.

Certain events during the task triggered the visual feedback GUI to send specific values to the operating GUI, through LSL, to trigger different components of the real-time BCI structure. Notably, trigger value 8 signaled the beginning of each trial, trigger value 6 indicated the initiation of the movement preparation period (as described in section 2.2.2), and trigger value 7 marked the conclusion of each training block (set of five trials). Furthermore, the visual feedback GUI communicated trial-specific information, including the trial name and number, directly to the operating GUI using a Transmission Control Protocol/Internet Protocol (TCPIP) connection.

##### 2.1.2.1 BCI structure

The BCI structure encompassed three primary processes:

1. *Initialization:* Upon initiation of data collection, an initialization function established four essential communication channels, illustrated in Figure 2. These channels included: (1) a link between BrainVision and LSL for real-time EEG streaming, (2) a pathway from Unity (visual feedback) to LSL for reception of trigger values and ankle angle in real-time, (3) a connection from LSL to MATLAB for the streaming of EEG and trigger values to the operating GUI, and (4) a direct link from Unity to MATLAB for transmission of trial information, such as the trial name. Following setup of these communication channels, LSL commenced recording and time stamping of EEG, trigger values, and ankle angle streams. Subsequently, a timer was initiated in MATLAB to execute the real-time BCI function every 50 ms, which retrieved the most recent trigger value and the latest 50 ms of the EEG stream from LSL.
2. *Real-time BCI:* During the neurofeedback training sessions, following the receipt of trigger 6, real time detection of Peak Negativity (PN) was initiated: the start of the prep bar (Unity trigger 6) was considered time 0. At 1,000 ms, we received inputs consisting of 64 channel × 25 (100 ms) packets of 50 EEG samples each. We then classified each sample, so our model outputted a series of 50 “0”s (rest) or “1”s (motor intent). If the detection model reached the number of 1 s as defined by a threshold value in any packet, it would trigger the NMES. EEG was epoched from 1,000 to 4,000 ms for training the LSTM model. The process started with packets 1–5, 500 ms after the start of the epoch (from time 1,000–1,500 ms). The actual detection started after packet 5 was received, at 1,500 ms after the prep bar started to move. The time between the receipt of packets 5–14 (1500–2,500 ms) should be designated by the detection process as rest. The participant is instructed to dorsiflex 3,000 ms after the start of the prep bar. As the PN detection window was considered 500 ms around this time (2,500–3,500 ms), packets 15–25 are associated with the PN detection window. If PN was detected and NMES triggered in packets 5–14, it was triggered during rest and was a false positive (FESfp variable); if PN was detected and NMES triggered within packets 15–25 (2,500–3,500 ms), it was triggered during motor planning and was a true positive. At that point (3,500 ms), we had an automatic NMES trigger if PN had not been detected and detection was therefore ended. We utilized the offline code to calculate false negatives which were defined as the failure to find at least one packet between numbers 15–25 with a total of 50 samples indicating motor intent. This process continued until trigger 7 (end of 5th trial in block) was received. Subsequently, the finalization function was triggered.
3. *Finalization*: This function halted the LSL recording and converted the recorded EEG data from the entire training block into EEGLAB’s preferred set format to facilitate post-hoc EEG analysis and generation of Event-Related Spectral Perturbation (ERSP) plots via the MATLAB-compatible EEGLAB toolbox (Delorme and Makeig, 2004) during data collection.

### 2.2 Data collection protocol

#### 2.2.1 Setup

The participant was seated in front of a monitor in a long sitting position on an examination table with the hip flexed approximately 30°, the knee supported in a small amount of flexion for comfort, and the ankle elevated slightly off the mat to enable unencumbered movement. For real-time tracking of ankle angle, the IMUs were placed on the top of the midfoot directly over the metatarsals and on the lateral side of the shank. The 64 EEG electrodes were positioned using the 5% 10–20 international system and actiCap (Brain Products) with FCz designated as the reference point. Conductive gel was inserted between the electrodes and scalp until the acceptable signal quality was achieved, which was indicated by a green LED on the electrodes. When the impedance between the electrode and the scalp was less than 20 kilo ohms, the LED on the EEG electrodes turned green.

#### 2.2.2 Training task and the visual feedback GUI

Each training block consisted of five trials of 10 s duration. At the beginning of each trial, the GUI prompted the participant to relax; this cue lasted 3 s. Subsequently, a horizontal red preparation bar (as illustrated in Figure 1) appeared on the screen to cue the subject to prepare to dorsiflex. The red bar was filled from left to right with a green bar which took 3 s. The participant was instructed to dorsiflex “as fast and far as possible” immediately after the green color reached the end of the bar. Once the movement execution phase began, the participant received real-time feedback of their ankle angle, represented as a vertical bar (angle bar). The participant was instructed to reach and attempt to surpass the target region at the top of the angle bar, which indicated 80 + % of their maximum dorsiflexion from the previous trial. The participant maintained the dorsiflexed position for 3 s until cued to relax again.

### 2.3 Motor intent detection

To identify an optimal detection method for our protocol we evaluated the performance of four different algorithms in detecting the motor preparation phase: (1) Thresholding, where a trial-specific threshold value was set in real-time using the mean of the Cz channel’s Event-Related Potentials (ERP) during the rest period of each trial; (2) Average PN, in which the mean timing of PN was determined during the calibration trials and then implemented during training (Mrachacz-Kersting et al., 2019) (not real-time); and two deep learning models both enabling real-time detection of brain activity; (3) a Multi-layer perceptron neural network (MLP-NN) (Behboodi et al., 2022), and (4) a Long-short term memory (LSTM) neural network. Machine Learning (ML) is a type of Artificial Intelligence (AI) that enables the analysis and synthesis of very large datasets, while Deep Learning (DL), a ML methodology, is a type of recurrent neural network especially proficient at extracting meaningful patterns from datasets and which is increasingly being utilized in healthcare settings (Aminizadeh et al., 2024). The proposed benefit of LSTM is to appreciably reduce calibration time when used repeatedly across sessions in an individual participant by utilizing and learning from their previous data over time, a feature which could be particularly advantageous when working with a pediatric population.

The parameters of each of these methods were adjusted during a calibration period. All of these algorithms utilized preprocessed EEG signals as input both during the calibration phase and for real-time BCI with the exception of Average PN.

#### 2.3.1 EEG preprocessing

The 64-channel EEG signals were band pass filtered to retain the MRCP frequency band (0.05 and 10 Hz). The signals were then spatially filtered to localize the prominent sources of brain activity and 20 channels situated over the sensorimotor cortex (FC5, FC3, FC1, FC2, FC4, FC6, C5, C3, C1, Cz, C2, C4, C6, CP5, CP3, CP1, CPz, CP2, CP4, CP6), involved with motor planning and execution, were extracted as the inputs for our movement intention detection algorithms.

#### 2.3.2 MRCP

MRCP (Mrachacz-Kersting et al., 2019) have been recognized previously as reliable signals for predicting movement intention (Mrachacz-Kersting et al., 2021). PN, a distinct feature of MRCP which typically occurs within 500 ms before movement onset, has been associated with the neurophysiological processes of motor planning and execution (Mrachacz-Kersting et al., 2016).

#### 2.3.3 Calibration

In each session, the initial five blocks of training, comprising 25 trials, were allocated for calibration of the detection model. For each trial, the preprocessed EEG data were segmented, or “epoched,” from 3 s before the participant’s cue to dorsiflex (initiation of preparation phase) until 1 s after the cue. To pinpoint the timing of PN for each trial, the minimum (maximum negative) values were computed during the movement preparation period, which extended from 500 ms prior to the cue to dorsiflex to 500 ms after the cue. This extended period was chosen because it allowed for potential variations in the onset of movement execution during the period when the PN is most likely to occur. Finally, all of the epochs from each channel were consolidated and averaged to generate the calibration ERP or MRCP signal.

#### 2.3.4 Real-time detection/BCI

##### 2.3.4.1 Threshold algorithm

###### 2.3.4.1.1 Calibration

The threshold for PN detection was computed using the mean and standard deviation for the first second of the rest portion of the calibration ERP from the central Cz electrode, characterized by a low likelihood of Peak Negativity occurrence. The ERP was then graphed alongside three threshold values represented as horizontal lines. These lines corresponded to 1, 2, and 3 standard deviations above the mean of the rest period. The appropriate standard deviation (Std) multiplier (Monge-Pereira et al., 2017; Kruse et al., 2020; Behboodi et al., 2022) for use during real-time BCI processing was visually identified.

###### 2.3.4.1.2 BCI

During neurofeedback training, preprocessed EEG data were fed into the algorithm every 50 ms (as detailed in section 2.1.2.1). If the mean of the Cz signal exceeded the threshold value chosen during the calibration phase, NMES was triggered. This algorithm used a Laplacian spatial filter.

##### 2.3.4.2 Average PN

In this algorithm, the average PN timing calculated during the calibration trials was utilized to trigger the NMES. During each trial, once the elapsed time calculated by the BCI timer reached the average PN timing, NMES was triggered (i.e., no real-time detection of PN). This open loop algorithm employed a Laplacian spatial filter as in the brain state stimulation system utilized by Mrachacz-Kersting et al. (2016) and Mrachacz-Kersting et al. (2019).

##### 2.3.4.3 Long-short term memory neural network

LSTM is a prominent type of recurrent neural network (RNN) renowned for its capability to learn both short and long-term temporal dependencies within time series data. Consequently, LSTM has extensive applications in sequence prediction scenarios. To discern minute temporal alterations within our preprocessed EEG data (our feature space) for the classification of participants’ intention to move, a solution that can memorize and subsequently accumulate these transitions is required. This demands the utilization of the inherent long and short-term memories in this deep learning model (Zhang et al., 2020).

###### 2.3.4.3.1 Calibration

**Figure 3:**
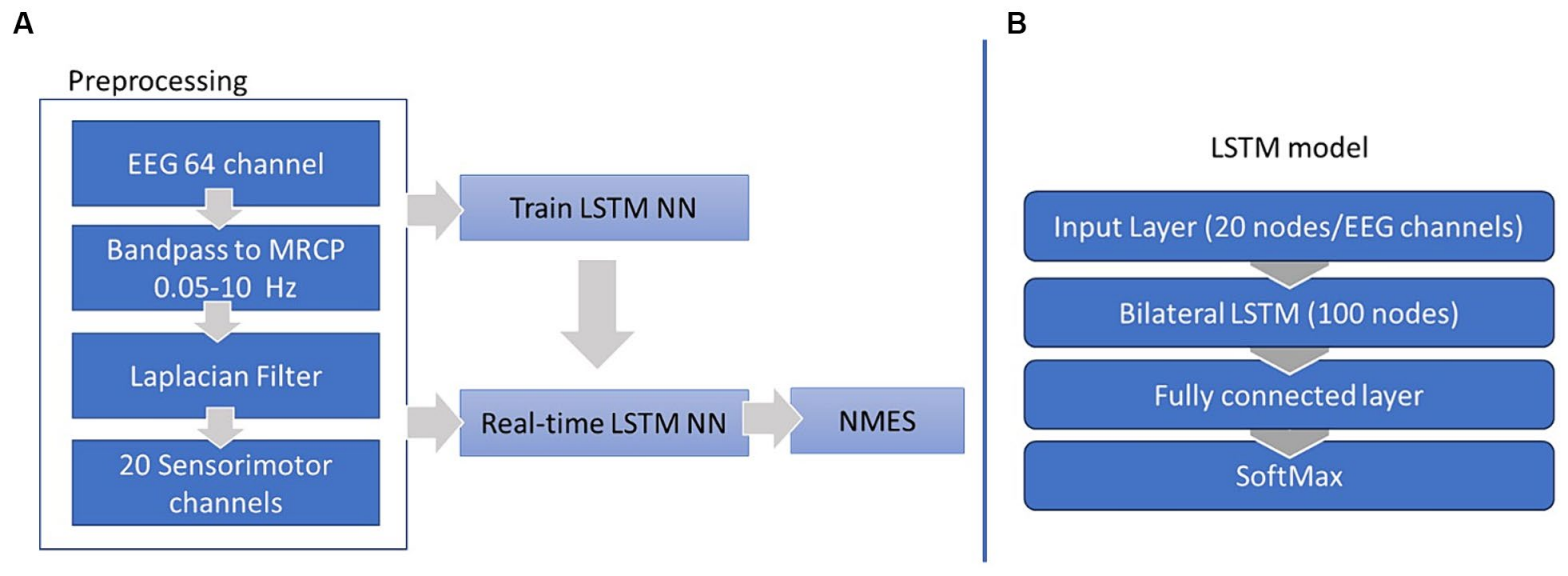
EEG preprocessing and LSTM training workflow **(A)** and LSTM structure **(B)**.

First, the preprocessed EEG were epoched into two classes, rest and motor intent (Figure 3). These epochs were labeled and used for the supervised training of our LSTM classifier. The classifier was constructed using the MATLAB Deep Learning toolbox and comprised four layers: an input layer with 21 nodes, each corresponding to one of the 20 EEG channels over the sensorimotor cortex; a bilateral LSTM layer consisting of 100 nodes (neurons) responsible for memorizing long and short term dependencies in the feature space; a fully connected layer (dense layer), a typical neural network layer; and a SoftMax layer serving as the output layer, responsible for generating probability distributions of our two classes (as illustrated in Figure 2B). The training process spanned 100 epochs and utilized the Adam optimizer.

###### 2.3.4.3.2 BCI

During the neurofeedback training, at each iteration of the BCI timer every 50 ms, a preprocessed EEG matrix, 64 (number of channels) by 25 (number of samples), was fed into the trained LSTM model. The model labeled each sample and generated an array of 25 binary elements, where 0 signified the “rest” class and 1 indicated the “motor intent” class. The sum of the elements within this array was then compared to a predetermined threshold, which in this case was set at 80% of the number of elements. If the sum surpassed this threshold, the EEG data sample was labeled “motor intent” and this triggered the NMES feedback.

##### 2.3.4.4 Multi-layer perceptron neural network

The MLP calibration and BCI procedure are similar to that of the LSTM; its structure, however, is different. Our proposed MLP consisted of two hidden layers, with a dimension of 20 at each layer (20 nodes); each node had ReLu activation function (Djamal et al., 2020). Unlike LSTM, the MLP was trained with the Stochastic Gradient Descent (SGD) optimizer (Craik et al., 2019) and a learning rate of 1. The preliminary results from the evaluation of this model are presented in Behboodi et al. (2022). The MLP structure was developed through a grid search approach. The number of batches, indicating the number of training time points (rows of the datasets) used per optimization step, and the number of hidden layers had minimal impact on the accuracy. Instead the accuracy was notably influenced by the layer dimensions, with a dimension of 20 for each hidden layer being identified as optimal. Additionally, a comparison of the Adam and SGD optimizer revealed a marginal effect on the accuracy. The learning rate of the optimizers, on the other hand, had a significant influence, with lr = 0.001 and lr = 0.01 performing optimally for Adam and SGD, respectively. Our final model utilized three hidden layers with 20 dimensions, a batch size of 8, a learning rate of 0.01, and the SGD optimizer.

### 2.4 Participants

As a first step, the overall system performance was evaluated on eight healthy adults. Additionally, the detection algorithms were tested using the EEG data collected from this group. Based on the evaluation results, we selected the best performing algorithm for use in our clinical study in children with cerebral palsy (CP).

Subsequently, we present here a case report from the first participant with CP utilizing the finalized BCI-NFT system in a 10-session protocol designed to train better selective control of ankle dorsiflexion, to demonstrate the system’s performance and the feasibility of our study protocol and to present some preliminary results in our target population.

Our protocol for both testing and implementing the system was approved by the NIH Institutional Review Board (#13-CC-0110), and all adult participants provided informed consent, with parent consent and child assent given for our participant with CP.

#### 2.4.1 Healthy adult cohort

Eight healthy subjects (6 females; age: 27.3 ± 7.1 years, [22.7–39.9]) with no history of neurological disease were enrolled in this study. This group participated in a single session of an ankle dorsiflexion training protocol using the BCI-NFT system to ensure that it functioned as intended and also to accumulate an EEG dataset for algorithm evaluation. At this developmental stage, NMES feedback was administered to the operator’s forearm rather than to the participant’s TA muscle which allowed the operator to assess whether the feedback activation occurred approximately at the intended time.

##### 2.4.1.1 Development of the detection algorithm

While the Average PN algorithm had shown promise in previous clinical trials (Mrachacz-Kersting et al., 2016, 2019), it was not considered a BCI system (Mrachacz-Kersting et al., 2021), as it employed a predetermined timing in an open-loop manner to trigger stimulation during the neurofeedback sessions rather than real-time detection. Our aim, however, was to develop a real-time detection algorithm capable of handling the individual and trial-by-trial variability in PN timing. Nonetheless, the Average PN algorithm was utilized as a reference for evaluating the performance of two deep learning algorithms, MLP-NN and LSTM models. The detection accuracy of the MLP-NN and LSTM were first compared to each other and then the chosen algorithm was compared to the Average PN algorithm. For each deep learning model a fivefold evaluation was conducted using the first 50% of the trials for training the model and the rest for testing the accuracy.

Finally, to optimize the detection of our deep learning model, we conducted two analyses:

1. Data labeling analysis: To train our deep learning model, it was necessary to label each calibration trial as “rest” or “motor intent.” We employed two different data labeling approaches: time-based and PN-based. *Time-based labeling* used the dorsiflexion cue, specifically the time when the preparation bar became fully green, as the reference point for labeling. One second around this reference point was designated as the “motor intent” class, while the second preceding it was marked as “rest.” *PN-based labeling* utilized the timing of PN in each trial as the reference point for data labeling. One second around the trial-specific PN time was assigned as the “motor intent” class, and the second preceding it was categorized as “rest.”
2. Spatial filtering analysis: We conducted an analysis to assess the impact of commonly used spatial filters for source localization, including Independent Component Analysis (ICA) and Laplacian on the accuracy of the deep learning models.

##### 2.4.1.2 Outcome measures for the healthy adult cohort

**Figure 4:**
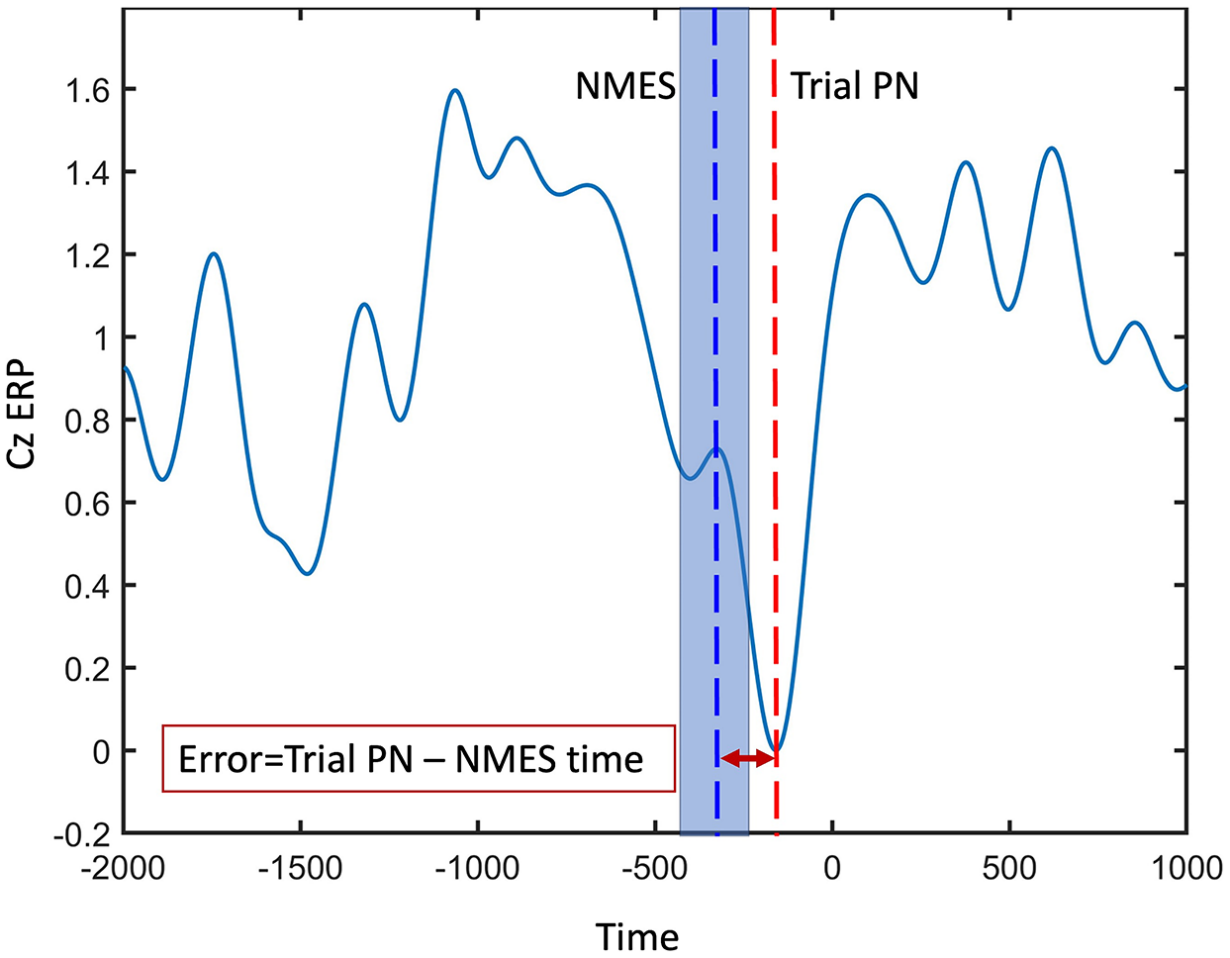
Peak negativity (PN) error was computed as the temporal difference between the PN of each trial (red dashed line), representing the desired stimulation time, and the instant at which the model activated the NMES (blue dashed line). The shaded blue area around blue dashed line represents the potential variability in detecting each trial’s PN.

Accuracy is commonly defined as the ratio of correctly labeled EEG data samples to the total number of detections within the PN detection window, reported as a percentage. The commonly used accuracy metric for quantifying the performance of the real-time models, as defined here, is not relevant for the Average PN algorithm. Thus, to assess and compare the chosen real-time model’s performance against the well-established Average PN, we utilized a detection error analysis. Error was computed as the temporal difference between the PN of each trial, representing the desired stimulation time, and the instant at which the chosen real-time model activated the NMES (Figure 4). Trial PN was defined as the absolute minimum of the preprocessed EEG within the detection window, i.e., 500 ms before the cue to move and up to 500 ms after the cue, in a virtual Cz channel (VCz). The VCz channel was generated by averaging EEG channels C1, C3, and Cz, located medially and centrally above the motor cortex. The detection error was presented as the mean ± Std and root mean squared error (RMSE). Furthermore, accuracy of the selected real-time algorithm was optimized under two conditions: data labeling and spatial filtering. To evaluate the statistical significance of the observed differences in the detection performance of the models, a two-sample t-test was performed, comparing the mean accuracies of the selected real-time LSTM model with the established Average PN approach, employing a significance level of *p* < 0.05.

#### 2.4.2 Participant with cerebral palsy

The goal of our BCI-NF training study is to improve selective control of ankle dorsiflexion in children with CP. This is a common impairment in those with either unilateral or bilateral CP. The protocol was to train the most affected ankle for each participant. Our first participant enrolled and completed the study in the summer of 2023, and we report the results here to demonstrate the robustness of our system as designed, illustrate the feasibility of the protocol for children with CP, and provide preliminary data on each of the functional and neural outcome measures for this participant.

The first participant was a 11.5-year-old boy with right unilateral cerebral palsy, classified as Gross Motor Function Classification System (GMFCS) level I. The etiology of his brain injury is not certain. His MRI indicated that he had diffuse white matter injury in the periventricular region typically consistent with preterm birth, even though he was born without complications at 38 weeks plus 5 days, and interestingly showed a focal injury in the right frontal region. However, his motor symptoms are mild and primarily isolated to his left upper and lower limbs. His main functional complaint relevant to this study was that it was difficult for him to run quickly because he tended to drag his right toes/forefoot. He was playing in a community baseball league and wanted to be able to run the bases faster.

##### 2.4.2.1 Training protocol

**Figure 5:**
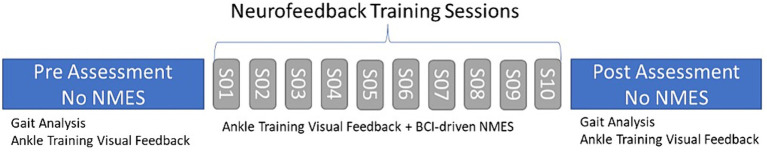
Training regimen for the participant with CP. We conducted two assessment sessions, pre- and post-training, and 10 training sessions.

He participated in a 10-session training regimen (Figure 5) during which he performed a minimum of 20 blocks of 5 dorsiflexion attempts, or 100 dorsiflexion trials, per session.

Because our selected deep learning model hinges on an adequate amount of training data, the initial calibration session required data from 50 trials, or 10 blocks of 5 repetitions each. Subsequently, the model was trained with data from the 50 calibration trials from the initial calibration or 100 trials from the previous training session, and an additional 25 trials from each day’s training session.

The task performed during each training session closely resembled the one described in section 2.2.2. with the notable additions that the participant received a performance score, in addition to ankle angle visual feedback during each trial and would receive electrical stimulation to the right TA activated by the EEG PN prior to each movement’s onset. For the training, we set a target goal of at least 80% of his mean maximum active ankle dorsiflexion angle during the calibration trials and asked the participant to try to exceed that on each trial. A vertical bar provided real-time feedback on ankle dorsiflexion angle recorded from the IMUs with the target range indicated and participant performance presented in real-time. The stimulation electrodes were positioned on the participant’s TA, and the stimulation intensity was adjusted in accordance with his comfort and tolerance levels. The stimulation pulse width and frequency remained constant at 150 μs and 40 Hz, respectively. However, we modulated the stimulation amplitude from 0 and incrementally increased it until reaching the maximum level that the subject found comfortable. Once determined, this amplitude was kept constant for the remainder of the session. This process was repeated for each session. The chosen frequency range and the pulse width fall within the standard parameters used in NMES research targeting the lower limb muscles of children with CP (Kim and Lee, 2016; Mooney and Rose, 2019).

##### 2.4.2.2 Assessment sessions

We conducted two assessment sessions, pre- and post-training. For these assessments, reflective markers were attached to anatomical landmarks on the participant’s lower limbs using a modified Helen Hayes marker set. Active ankle dorsiflexion range of motion when seated as well as over ground gait kinematics when walking at freely-selected and fast speeds were recorded using a 12 camera 3D motion capture system (Vicon, Denver CO). The goal was to assess whether any potential improvements in ankle motion from the training would transfer to improvements in ankle motion during gait. While the motion capture markers were used to record ankle angle, the visual feedback for the ankle joint angle was based on IMU recordings.

##### 2.4.2.3 Outcome measures

The primary motor outcome measures were ankle dorsiflexion angle and joint angular velocity during task execution and during over ground free and fast speed walking. We also computed temporal–spatial gait measures (velocity, cadence and step length). Additionally, system detection accuracy for EEG PN was assessed for the 10 training sessions to compare performance in CP to that of healthy adults. The primary neural outcome measure was the magnitude of event-related desynchronization (ERD) in the alpha (8–12 Hz) and beta (13–30 Hz) bands in comparable motor-related brain regions before and after training. We also assessed a laterality index for brain regions with ICs in both hemispheres for both timepoints. This was calculated as: (ERD on hemisphere contralateral to trained side – ERD on ipsilateral side)/ (ERD on hemisphere contralateral to trained side + ERD on ipsilateral side).

##### 2.4.2.4 Transfer learning

To enhance user compliance and maximize the number of neurofeedback trials, we aimed to reduce the duration of each training session, eventually achieving a timeframe of less than 2 h, including the setup time. The entire training protocol including set-up time, calibration trials, up to 100 motor attempts during training and ample rest time to minimize muscle fatigue or to take snack breaks never exceeded 3 h. For our pediatric population with CP which is more likely to experience muscle fatigue and become bored with performing the same task repetitively, we decided to utilize a machine (transfer) learning algorithm here which can appreciably reduce the number of calibration trials once the initial model is built. The primary goal was to pre-train a model using all of the trials of the previous session, and subsequently fine-tune it during the calibration phase of the current session, as opposed to training an entirely new model for each session. This enables them to devote more of their effort and attention on the motor training itself. The session-by-session accuracy of the transferred LSTM model was calculated and compared with the accuracy of the non-transferred model trained on the 50 initial trials of each session.

##### 2.4.2.5 EEG data analysis

**Figure 6:**
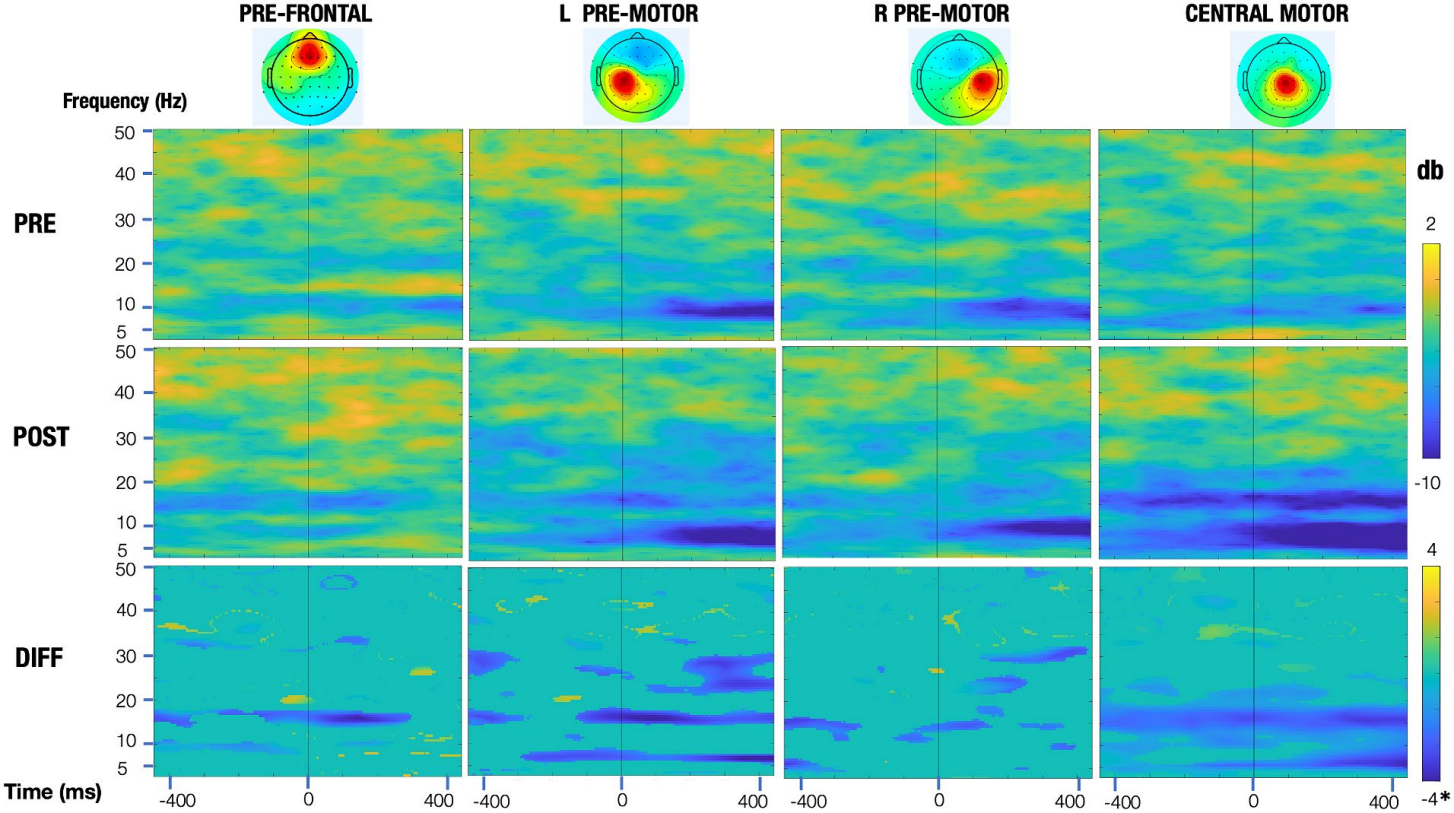
Event-related spectral perturbation (ERSP) plots of the motor regions pre and post training (top and middle row, respectively) and their significance masked differences (bottom row). All rows share a common time and frequency axis, with time zero corresponding to dorsiflexion onset of the trained leg (vertical line). The top two rows (pre and post) share a common colorbar (change in power in decibels), with blue representing a decrease in power relative to that session’s baseline and red representing an increase in power. For the bottom row, the significance masked ERSPs (green means a voxel was not significant at an alpha value of 0.05) are scaled symmetrically from −4 db to 4 db*, with the exception of the central motor region, which is scaled from −8 to 8 db, to adequately represent the larger relative changes seen in this region.

EEGLAB open-source software was utilized, with a processing stream detailed in an earlier publication (Hinchberger et al., 2023). Briefly, steps included attenuation of power line noise using the EEGLAB cleanline function, automatic removal of bad channels using the clean_rawdata function, creation of a merged file with all rest and experimental trials, visual removal of noisy periods, down-sampling to 250 Hz, application of Artifact Subspace Reconstruction (ASR) (Mullen et al., 2013), re-referencing to a common-average, and 1 Hz high-pass filtering. Adaptive Mixture Independent Component Analysis (AMICA) was used to parse mixed scalp signals into maximally temporally independent component (IC) signals. The EEGLAB DIPFIT algorithm was used to generate a best-fit equivalent current dipole for each IC, removing those with residual variance >20% or topographical sparseness >5. Scalp topographies, power spectra, and dipole locations were evaluated, and those deemed of non-cortical origin were removed. Selected epochs were from 1 s before to 1 s after movement onset. Rest trials from each assessment were used as the baseline which was subtracted from the movement trials for that assessment. To demonstrate significant differences across assessment sessions, we created significance masked ERSPs using the EEGLAB “condstat” function, 200 bootstrapped surrogate datasets, and a significance value of less than 0.05. Non-significant values were set to 0 (green) in the difference ERSPs (row 3, Figure 6).

## 3 Results

### 3.1 Healthy adult cohort

In this study, we assessed the performance of various detection algorithms using EEG datasets collected from healthy participants. The thresholding algorithm was excluded from further analysis due to its frequent misdetections, often stemming from threshold values set during calibration that were too high for real-time training trials, hindering effective triggering of NMES.

#### 3.1.1 Comparing the deep learning models: MLP vs. LSTM NN

The mean detection accuracy of LSTM across healthy participants (88% ±10) was significantly higher than that of MLP-NN (85% ± 8), *p* < 0.001. The modest difference in mean accuracy led to the selection of the LSTM model. More importantly, LSTM was selected due to its ability to handle subject heterogeneity in individuals with CP by leveraging its capacity to process long-term and short term interdependencies in a time-series, as its name implies.

#### 3.1.2 Comparing the selected deep learning LSTM model to the average PN algorithm

Although the RMSE value of 220 ms for LSTM was not significantly different from that of the Average PN algorithm (214 ms, *p* = 0.343), LSTM mean error was negative (−70 ms ± 150), whereas the Average PN algorithm’s mean error was positive (128 ms ± 180) meaning that on average LSTM detected the PN sooner than the average PN. This analysis demonstrated that LSTM has comparable performance to the Average PN algorithm, however, LSTM can be incorporated in a BCI system and it can be implemented with greater flexibility in parameters optimization increasing accuracy in those with neurological injuries who may have greater individual variability in PN occurrence and timing.

#### 3.1.3 Data labeling

The model was significantly more accurate under time-based labeling (88% ± 10) than PN-based labeling (83% ± 11), *p* < 0.001.

#### 3.1.4 Spatial filtering

Despite being significantly different, the accuracy of the LSTM with a Laplacian spatial filter was not appreciably higher than that of an ICA spatial filter (88% ±10 vs. 85% ±11, *p* < 0.048). The main reason we opted for the Laplacian filter over ICA was its faster operation, as ICA could take up to 10 min depending on its parameters. Moreover, ICA has the potential to alter the polarity of MRCP, which may impact detection reliability. Additionally, the selection of the appropriate component in ICA requires visual inspection which is not needed with Laplacian.

### 3.2 Participant with CP

The system performed well throughout all assessments and training without any major issues. The participant was able to complete all needed calibration trials and perform the desired 100 trials per training session. During the first training session, we noted that our participant was not responding in a timely manner to the cue to move, which would delay the EEG signal needed to activate the NMES beyond the time window we had allotted. Therefore, for several of the subsequent blocks during the first session, we asked him to focus on reducing his reaction time, i.e., to attempt to move as soon as he was cued to do so. He quickly improved on this aspect and retained good timing across all training sessions. It was also noted on reviewing his performance during his first training session that he had a functional range (greater than 10°) of dorsiflexion. Therefore, for the rest of training sessions, we instructed him to focus more on velocity while still reaching his target dorsiflexion angle for each session.

#### 3.2.1 Motor outcome

Motor outcomes data are summarized in Table 1, including the mean active ankle dorsiflexion angle while seated, peak dorsiflexion angle and joint angular velocity during initial contact and the swing phase of gait across 20 strides in the free and fast walking trials with associated *p*-values, and temporal–spatial gait measures. Maximum dorsiflexion did not increase in any condition, and where significant, a small decrease was shown. However, there was a very large increase in dorsiflexion velocity after BCI-NFT training which, while significant, did not transfer to walking. Interestingly though, gait speed, and its components, cadence and step length, all increased after the training, with a significant difference in right step length for the freely-selected speed condition.

**Table 1:** Temporal–spatial gait measures and ankle dorsiflexion (DF) measurements pre- and post-training for the participant.

Outcomes	Right pre	Left pre	Right post	Left post	Right diff	Left diff	Right p val	Left p val
Gait speed free (m/s)	1.19		1.34		0.15			
Cadence (steps/min)	120.1	132.9	125.6	139.3	5.54	6.40		
Step length (m)	0.56	0.57	0.61	0.61	0.05	0.04	**0.001**	0.054
Gait speed fast (m/s)	1.70		1.84		0.14			
Cadence fast (steps/min)	138.3	153.1	148.0	157.4	9.68	4.34		
Step length fast (m)	0.72	0.69	0.72	0.73	0.001	0.04	0.19	0.08
MAX DF angle swing (°)	4.58	9.90	1.43	6.90	−3.15	−3.00	**0.003**	**0.001**
DF angle initial contact (°)	−2.70	0.88	−2.86	−1.15	−0.16	−2.03	0.48	**0.032**
MAX DF velocity stance (°/s)	141.3	110.3	146.9	151.9	5.69	41.6	0.48	**0.001**
MAX DF velocity swing (°/s)	121.6	199.4	115.0	191.3	−6.67	−8.03	0.62	0.36
MAX DF angle in sitting (°)	10.8		8.46		−2.37		**0.001**	
MAX DF velocity in sitting (°/s)	133.4		234.0		100.6		**0.001**	

The right side was trained, so the greatest differences were anticipated on that side. BOLD = *p* < 0.05.

#### 3.2.2 EEG analysis

Event-related spectral perturbation (ERSP) plots pre- and post-training are shown in Figure 6 for the right and left pre-motor/supplementary motor regions and for the central motor region that is localized just to the right of midline. These plots demonstrate that the greatest change in activation was in the central motor region, with beta differences being more prominent. Additionally, mean event-related desynchronization (ERD) values are presented in Table 2. To calculate ERD, EEG was epoched from 1 s before to 1 s after dorsiflexion movement onset. EEG was recorded during active dorsiflexion trials while seated and included data from motor-related brain regions. These ERDs indicate the amount of power reduction from rest in the alpha and beta bands pre- and post-training.

**Table 2:** Mean event-related desynchronization (ERD) values indicating the amount of power reduction from rest in the alpha and beta bands in motor-related brain regions pre-and post-training, recorded during active dorsiflexion trials while seated.

Outcome	Right pre	Left pre	Right post	Left post
Prefrontal alpha	1.61		3.67	
Prefrontal beta	2.60		3.35	
Premotor region alpha	5.44	6.01	6.29	6.05
Premotor region beta	3.56	3.68	4.15	5.11
Central motor alpha	4.83		7.63	
Central motor beta	3.18		5.45	

#### 3.2.3 Transfer learning

**Figure 7:**
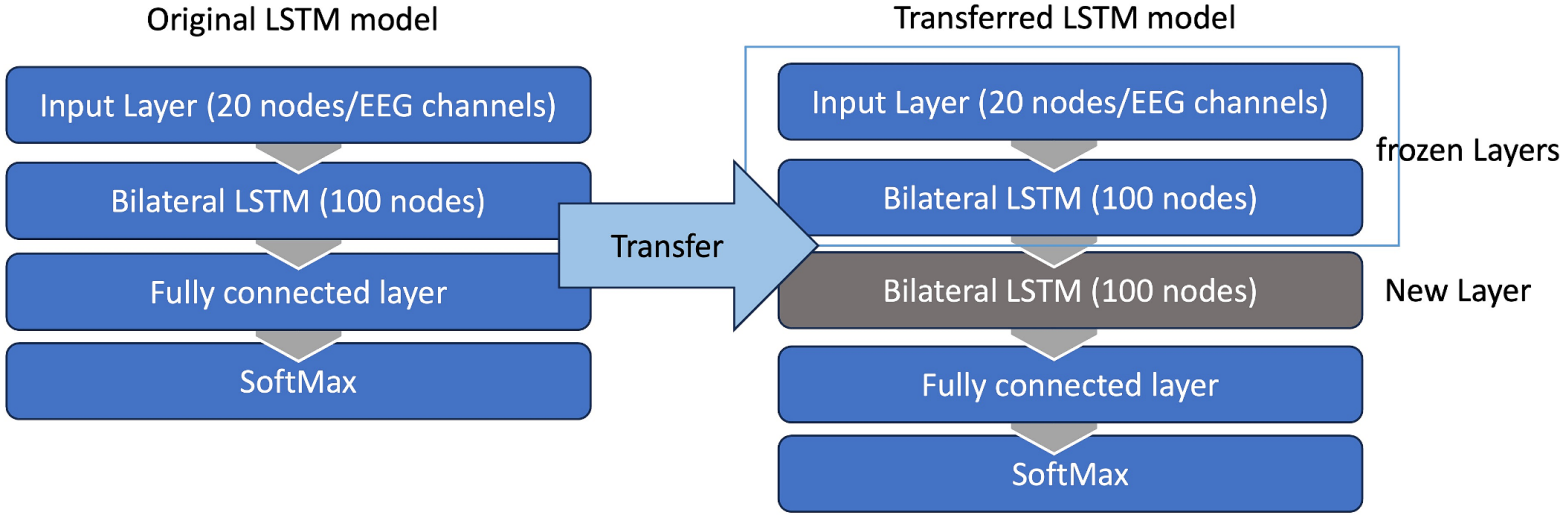
Transfer learning. The LSTM model was trained with trials collected during the previous session (100 trials) and then transferred to the current session using the first 25 trials. The weightings of the first two layers of this LSTM model were held constant, or “frozen,” then a new bilateral LSTM layer was inserted to model. Due to the frozen state of the first two layers only the weights of the new bilateral LSTM and the fully connected layer were updated during the transfer learning.

**Figure 8:**
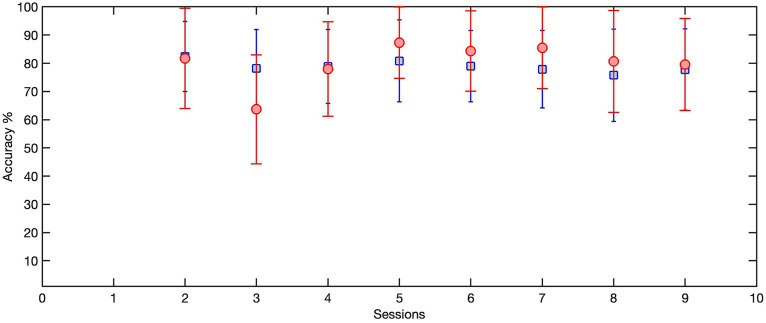
The session-by-session accuracy of the transferred LSTM, trained on 100 trials from the previous session and transferred to 25 trials of the current session (red circles), compared with the accuracy of the LSTM model if it was not transferred and instead was trained on the first 50 trials of each session (blue squares). Here we only included the sessions that used transferred LSTM as the detection model.

Given that the performance of any deep learning model hinges on an adequate amount of training data, this strategy ensured a more efficient utilization of available data while minimizing calibration time. Consequently, the LSTM model was trained with the trials collected during the previous session (over100 trials) and then transferred to the current session using the first 25 trials. To this end, the weightings of the first two layers of this LSTM model were held constant, or “frozen,” then a new bilateral LSTM layer with 100 nodes was added to the structure (Figure 7). Due to the frozen state of the first two layers, only the weights of the new bilateral LSTM and the fully connected layer were updated during transfer learning. To minimize the impact of session-by-session variability in our feature space (preprocessed EEG) we employed Z-score normalization at the input layer, i.e., the input EEG was scaled by subtracting its mean and dividing by its standard deviation. In the initial session, the model underwent training using data from 50 trials. Figure 8 demonstrates the session-by-session accuracy of the transferred LSTM compared with that of the LSTM model if it was not transferred, i.e., if it was trained on only the 50 initial trials of each session. The mean detection accuracy of the transferred model was 80.8% vs. 78.9% without transfer learning (*p* = 0.064). Detection of the wrong signal as the PN occurred in about 17% of trials (false positives) with false negatives being rare. The session-by-session accuracy results are in Table 3. Please note that the accuracy results were processed post-hoc using the model that was trained at each session of the data collection.

**Table 3:** Detection accuracy showing that on average 80% of trials detected the peak negativity in the proper window and activated the stimulator.

Session	False positives (%)	False negatives (%)
2	19.1	0
3	10.8	1.5
4	26.2	0
5	15.4	1.5
6	17.5	3.2
7	10.3	1.7
9	19.1	1.5
10	19.1	6.4
Mean	17.2	2.0

Only the sessions that used transferred LSTM (sessions 2–10) as the detection model were included.

## 4 Discussion

To extend the application of BCI-NFT from the stroke population to a pediatric population with CP, we designed a MA-based BCI-NFT to train ankle dorsiflexion in children with CP. The BCI-NFT system employed LSTM to detect PN in MRCP, an EEG feature generated by an attempt to dorsiflex the ankle in real-time, and thereby activate enriched sensory feedback to the TA muscle by application of NMES, in an associative learning paradigm. The system was finalized using a cohort of eight healthy individuals and evaluated on a child with CP in a 10-session dorsiflexion training protocol. Considering the heterogeneity of cortical activity in the CP population and the location of the ankle on the somatotopic motor map of the primary motor cortex (M1), the system demonstrated good to high reliability in applying NMES feedback, as demonstrated by the reported false positives and false negatives that may have also been affected by some expected fluctuations in attentiveness to the task given the multiple repetitions required.

### 4.1 BCI-NFT system accuracy

Similar to other BCI systems, one of our major considerations in designing our system was to achieve as high a detection accuracy of motor intent as possible, such that the task is assisted and sensory feedback enhanced only during focused efforts to perform the task, and not when the participant may not be attending as well to the task. Detection accuracy is especially important in BCI-NFT systems as false positives may lead to mal-adaptive neuroplasticity by potentially enhancing inappropriate associations or failing to promote appropriate ones. Previous MA-based BCI-NFT systems predominantly used machine learning classifiers such as linear LDA (Ono et al., 2013; McCrimmon et al., 2014; Mukaino et al., 2014; Osuagwu et al., 2016; Chen et al., 2020), SVM (Chowdhury et al., 2018; Bhagat et al., 2020; Chowdhury et al., 2020), Gaussian (Biasiucci et al., 2018), and logistic regression (Ibáñez et al., 2017). Our study uniquely employed LSTM (a deep learning algorithm often utilized in BCI applications), for detecting motor intent, demonstrating the potential for enhanced accuracy in MRCP detection. The performance of motor attempt BCI-NFT systems designed for motor rehabilitation in neurological populations has been primarily characterized by detection accuracies ranging from 70 to 80% in the stroke population. A requisite for effective BCI-NFT, as established in MI BCI-NFT systems, is the attainment of a detection accuracy exceeding 70% (Vidaurre and Blankertz, 2010). The implementation of a Bayesian classifier in a MI BCI-NFT protocol for children with CP by Bobrov et al. demonstrated a maximum 70% detection accuracy (Bobrov et al., 2020). In our study, the BCI-NFT system showcased a robust detection accuracy of over 88% in the healthy cohort. However, our participant with CP exhibited a slightly reduced detection accuracy of 79%, but still surpassing the 70% threshold and similar to studies in other populations with and without neurological conditions.

Among notable studies employing real-time MRCP feature detection, Bhagat et al. reported a 79% detection rate in 10 participants post-stroke, alongside a 23% false positive rate, utilizing 15 electrodes over the sensorimotor cortex (SMC) and an SVM classifier (Bhagat et al., 2020). Xu et al. introduced a manifold learning algorithm followed by LDA, demonstrating a detection delay of 200–400 ms and a detection accuracy exceeding 80% for the classification of motor intent in healthy individuals (Ren et al., 2014). Additionally, Niazi et al. introduced an optimal spatial filter, reducing detection timing by 66.6 ± 121 ms, accompanied by a detection accuracy of 82.5% (Niazi et al., 2013). Lawhern et al. contributed significantly to the utilization of deep learning for detecting MRCP features, evaluating EEGNet, a convolutional neural network (CNN), in detecting motor intent during index finger tapping in 13 healthy individuals, with a within-subject accuracy of approximately 80% (Lawhern et al., 2018). It is important to note that all of these studies reporting BCI performance focused on upper limb movement attempts, which involve a larger more superficial area of the sensorimotor cortex, which is more easily accessible with EEG.

### 4.2 Case discussion in a participant with CP

Our neurofeedback system worked exactly as intended for all assessments and training with our first participant with CP. He was initially not as attentive to the cued movement timing as desired, but he responded well once prompted to react in a more timely manner. The ability to train dorsiflexion range of motion and/or velocity is an advantage of our design that was helpful for this particular child who needed to practice keeping his foot more dorsiflexed at key points in the gait cycle but who could also potentially also benefit from improvements in dorsiflexion velocity. Changes were predominanetly task specific, i.e., they were the greatest for dorsiflexion velocity, which was the primary focus of his training sessions, but only during the training task. Improvements in temporal–spatial gait parameters, while encouraging, could not be explained by positive changes in ankle dorsiflexion range or velocity during gait, which were not found.

The differences in detection accuracy between healthy adults and a child with CP are not surprising. Maintaining attention and motivation was more challenging in this younger child than it was in adults, and we believe that inattention may account at least in part for his lower detection accuracy. For future participants, we do not plan to provide stimulation if the PN is not detected in the detection time window, so that they are not reinforced in a similar manner when they are not as engaged in the task, because inappropriate stimulation may have a negative effect on neuroplasticity by associating inappropriate behavior with enhanced sensory feedback.

The EEG results showed minimal changes post-training in brain regions involved in motor planning, with the exception of small changes mainly in the beta band in the untrained hemisphere, which may indicate a need to recruit ipsilateral pathways to improve performance. The most dramatic improvement was in the central motor region in the beta band. While this IC was localized slightly to the right of midline, the position of the ankle area within the central sulcus makes determining hemispheric location difficult at best, although it is also possible that this could reflect reorganization due to his left brain injury.

While far more data are needed regarding the immediate and long-term effects of this training in CP before any generalized conclusions can be made, the functional changes reported here after only 10 sessions are encouraging. Surprisingly, fatigue did not appear to be a limiting factor during the training. This participant’s only complaint was of boredom due to the number of repetitions requested. Future plans are to gamify the training so that it is more motivating and enjoyable. Data collection on additional participants is currently underway.

### 4.3 Future plans

In our BCI-NFT system, we successfully employed LSTM as a classifier for motor intent. In the future, we aim to utilize LSTM’s predictive capabilities, specifically focusing on predicting future states within EEG time series to forecast PN occurrences, thereby enhancing the system’s adaptability to the heterogeneity of cortical activity in children with CP. Additionally, our strategy involves the integration of reinforcement learning techniques to enhance the performance of our BCI in accurately predicting or detecting motor intent states. Our first participant with CP exhibited fairly good motor control in that he could dorsiflex repeatedly on command. Children with CP can have a wide range of impairments, and future participants may have greater difficulty reliably performing the task, may not be able to move the ankle at all, or may be prone to fatigue with repeated attempts. These situations would likely present additional challenges to our current system and its performance and effectiveness that we have not yet addressed here. We also plan to collaborate with groups who have commercialized these systems so that they can be utilized in clinics and even in patients’ homes, assuming that BCI-NFT demonstrates efficacy and efficiency in improving motor function in CP.

## 5 Conclusion

BCI-NFT systems utilizing motor attempt and newer algorithms that can reduce calibration time while maintaining accuracy provide a feasible method for motor training in children with CP with the possibility of accelerating improvements in motor performance as well as neuroplasticity with stronger evidence of effectiveness needed before implementation in clinical settings.

## Data availability statement

The raw data supporting the conclusions of this article will be made available by the authors, without undue reservation.

## Ethics statement

The studies involving humans were approved by the National Institutes of Health Institutional Review Board. The studies were conducted in accordance with the local legislation and institutional requirements. Written informed consent for participation in this study was provided by the participants’ legal guardians/next of kin. Written informed consent was obtained from the individual(s), and minor (s)’ legal guardian/next of kin, for the publication of any potentially identifiable images or data included in this article.

## Author contributions

AB: Conceptualization, Data curation, Formal analysis, Investigation, Methodology, Project administration, Software, Writing – original draft. JK: Data curation, Investigation, Methodology, Writing – review & editing. AG: Conceptualization, Methodology, Software, Writing – review & editing. CP: Investigation, Methodology, Software, Writing – review & editing. SP: Investigation, Project administration, Writing – review & editing. DD: Conceptualization, Formal analysis, Funding acquisition, Investigation, Methodology, Project administration, Resources, Supervision, Writing – original draft, Writing – review & editing.
